# Potential for the specialty of Family Medicine in Botswana: A discussion paper 

**DOI:** 10.4102/phcfm.v4i1.352

**Published:** 2012-10-31

**Authors:** Luise Parsons, Taatske Rijken, Deogratias O. Mbuka, Oathokwa Nkomazana

**Affiliations:** 1 Department of Family Medicine, University of Botswana, Botswana

## Introduction

Family Medicine is developing rapidly as a medical and academic specialty in sub-Saharan Africa. The multifactorial policy drivers are not well described, but include population health needs, the World Health Organisation’s initiative^1^ to re-energise primary care in their 2008 report, failure of hospital services and even vertical programmes to cope with the continuing HIV epidemic in many sub-Saharan countries in Africa, and a growing middle class which expects more and better quality from primary care.^2,3^ These are but a few reasons why Primary Health Care and Family Medicine are enjoying a resurgence of interest, investment and recruitment.

Botswana is one of the latest countries in sub-Sahara Africa to open a School of Medicine (2009) and establish an Academic Department of Family Medicine (2010) to offer undergraduate and postgraduate training. The aim is to produce appropriately skilled generalist doctors who can function within and lead primary care to transform quality and access to health care. The curriculum is based on the regional definition of Family Medicine in an African context, articulated in the 2010 Statement of Consensus on Family Medicine in Africa.^4,5^ However, the debate has already begun within Botswana about whether this definition and our training programme are too Euro-centric and academic.^6^

It is expected that in Southern Africa, accredited family physicians will be competent to meet 90% of the health needs of communities in their designated districts.^7,8^ Meeting this challenging target with doctors drawn from and rooted in local communities who are familiar with local culture, language and traditions is the aim of Botswana’s new specialty of Family Medicine. This article explores these aspirations and the potential for Family Medicine in Botswana at the beginning of this new training programme.

## Background

Botswana is a land-locked country in Southern Africa with a land mass equivalent to France and Switzerland combined, or Texas, with a population of 2.1 million people.^9^ Granted independence in 1966, Botswana prides itself on having the longest unbroken history of democracy in Africa, and has been economically successful since the discovery of diamonds and other mineral resources in 1967. Indeed, it has developed from being one of the top 10 poorest nations in the world at independence to a middle-income country in the 21st century, and is hailed by some as one of the economic success stories of Africa.^10^ The Botswana Government has invested much of this wealth in education, health and other infrastructure such as roads and public buildings.

At independence in 1966, the British ‘Protectors’ left the former Bechuanaland with 3 km of tarred road, two public hospitals (in addition to largely mission-run hospitals) and one secondary school.

Since independence the Ministry of Health has sent nearly 1000 citizens overseas for undergraduate and specialist training in medicine. Unfortunately, in 2009 only slightly more than 60 of these individuals were back in the country, with a minority working in the public sector or at the University of Botswana. The key University of Botswana Senate document^11^ that successfully proposed the MMed programme quotes a 2005 World Health Organisation report which indicated that of the 720 doctors working full time in Botswana, only 10% were citizens. Until recently, not a single Setswana-speaking doctor practiced primary care in the rural and remote districts where around 90% of patients present for treatment in the healthcare system.

>The population’s health needs are pressing. Epidemiological statistics are not easy to find in Botswana, but the public health needs of the population are great. Table 1 from the Ministry of Health indicates progress in Botswana towards the United Nations Millennium Development Goals (selected indicators, 2007).

**TABLE 1:**
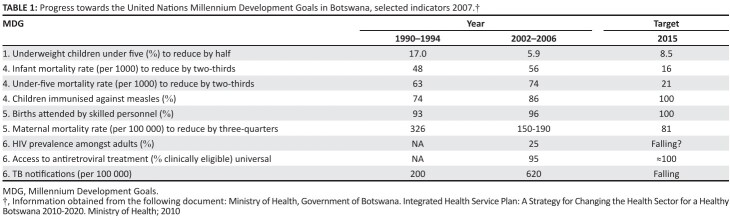
Progress towards the United Nations Millennium Development Goals in Botswana, selected indicators 2007.

The Integrated Health Service Plan 2010–2020^12^ identifies the main health-related issues in Botswana over the next 10 years as follows:

- high infant and child mortality, including post-neonatal mortality
- high maternal mortality
- high mortality and morbidity from communicable diseases (such as HIV and AIDS, diarrhoeal diseases, acute respiratory infections, etc.)
- malnutrition amongst women (obesity) and children (general and micronutrient under-nutrition)
-  high incidence of infectious diseases such as HIV and AIDS and tuberculosis
- increases in non-communicable diseases
- poor quality of care
- increases in injuries and accidents
- shortages of skilled human resources
- weak quality management and regulation in both public and private sectors
- unhealthy lifestyles and widespread inappropriate health-seeking behaviour
- weak sector-wide management

This was the context in which the Government supported a medical school at the University of Botswana. In 1995 a feasibility study recommended establishment of a medical school as a ‘long term endeavor to help meet the health care needs of the country’^11^ Based on these recommendations a 1998 Presidential Directive enjoined the University to commence the phased establishment of a School of Medicine. This Directive established a high-ranking committee responsible for overall planning and costing of this initiative. After many years of planning an Interim Founding Dean was appointed in 2006, and the undergraduate curriculum leading to MBBS was approved by Senate and Council in 2007. In 2010 the University of Botswana Senate agreed on proposals for Master of Medicine (MMed) programmes in the specialties of Paediatrics and Internal Medicine (first intakes January 2010); Family Medicine, Public health, Emergency Medicine and Anaesthetics (first intakes 2011).^13^ 

The Botswana Government’s Vision 2016 statement is as follows:

By the year 2016, all Batswana will have access to good quality health facilities, including both primary and curative services within reasonable travelling distance. Mental health treatment will be accessible to all … Botswana will be fully equipped and able to deal with unexpected epidemics, or the outbreak of new and hitherto unknown disease … If there is not at that time an affordable cure, all people who are suffering from AIDS related illness will have access to good quality treatment in the health facilities, community or the workplace so that they can continue to live full and productive lives for as long as possible ...^14^

In addition, the Ministry of Health has as a target that no citizen should have to travel more than 10 km to a healthcare facility.

**FIGURE 1:**
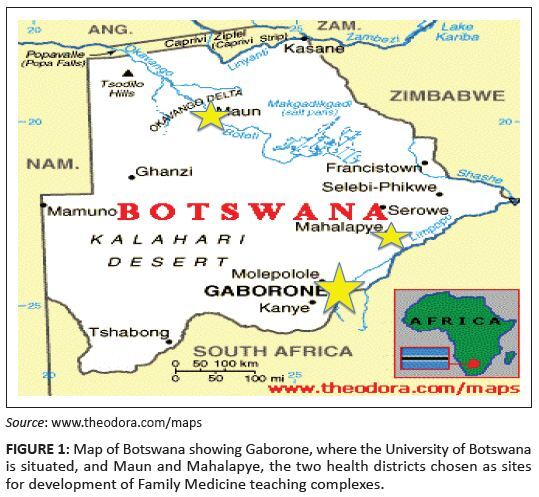
Map of Botswana showing Gaborone, where the University of Botswana is situated, and Maun and Mahalapye, the two health districts chosen as sites for development of Family Medicine teaching complexes.

The geography and demography of Botswana pose particular challenges to the development of primary care and Family Medicine. The population who most need Family Medicine Secialists (FMS) are largely in the rural areas. A decentralised training programme fits with the population health needs and a community-oriented primary care model of service.^15^ In this context and using evidence from elsewhere about the value of recruitment and retention of trained healthcare workers in remote and rural areas, a decentralised model of training FMS was advocated. Following scoping exercises by an external consultant^16^ in Maun and the newly appointed FMS17 in Mahalapye, these two health districts were chosen as the sites for development of Family Medicine teaching complexes (Figure 1). The organisation of Botswana’s health care system for 2007–2016 (Table 2).18 

**TABLE 2:**
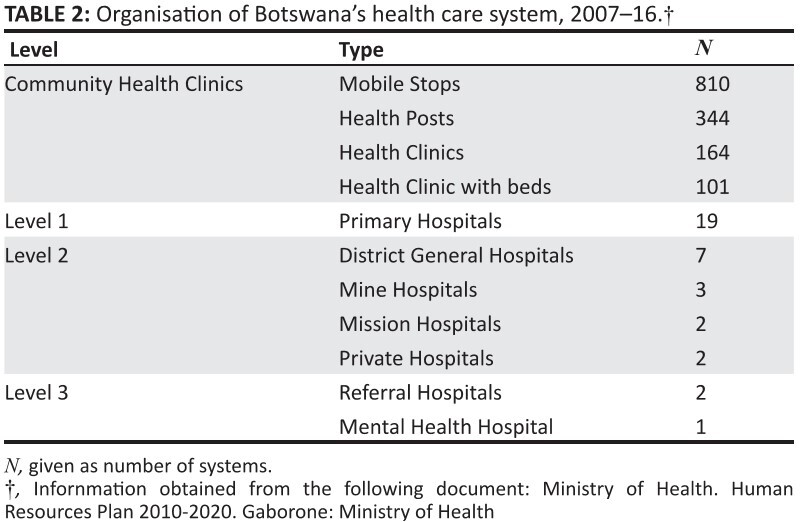
Organisation of Botswana’s health care system, 2007–16.

Outside the referral hospitals, medical services are currently largely provided by expatriate medical officers and specialists from, amongst others, Africa, Cuba, China and Europe. The Ministry of Health Integrated Service Plan for Botswana 2010–2020^12^ reports that in 2007, 240 of the 595 doctors in Botswana were expatriates (Table 3).

**TABLE 3:**
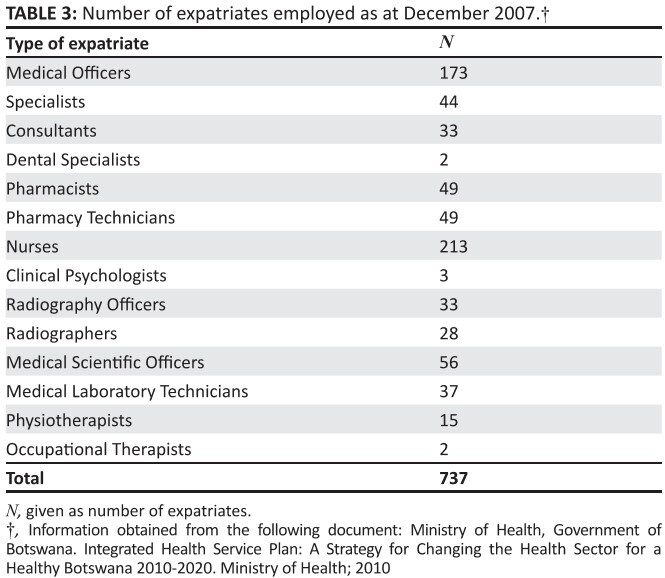
Number of expatriates employed as at December 2007.

These doctors vary in technical expertise, commitment and standards of professionalism, and may not speak much English (let alone Setswana), in communities where English is not widely spoken. In addition, doctors are inequitably distributed, with 60% working in Gaborone and Francistown where only 14% of the population live.^11^ Traditional medicine is often the first point of contact for sick people and their families, but to date there has been little experience of working with traditional healers.

Nurse training in Botswana has a long and successful history in the Ministry of Health (since 1978), and most nurses, especially those in leadership, are Batswana. Nurses are more equitably distributed, with only 11% working in the referral hospitals.^17^ This experience of the nurse workforce being more equitably distributed after training in decentralised Institute of Health Sciences is a model for Family Medicine.

Commitment to develop the specialty of Family Medicine was strongly supported by a seminal workshop convened in 2008 by the University of Botswana to scope the demand for Family Medicine and where it could be most effective. The workshop was attended by leaders from the Ministries of Health, Education and Local Government, the Botswana Health Professions Council (regulatory council) and regional and international experts in Family Medicine and primary care. The workshop was addressed by the Professor of Family Medicine at Stellenbosch University near Cape Town, South Africa. The key focus of this workshop was to assess the need for FMS in Botswana, and to discuss the models of training informed by the emerging Consensus on Family Medicine in Africa.^4,5^ 

Subsequently the Botswana School of Medicine has been twinned with the University of Stellenbosch for support with development of the Family Medicine programme. Funding for this vital support came from VLIR (the Flemish Interuniversity Council). This twinning arrangement has been instrumental in development of the Family Medicine programme and also the residential core modules. The University of Botswana and its partner Stellenbosch University also agreed to using the VLIR funding to buy into the Stellenbosch curriculum for the first two years. This has been essential whilst faculty members are recruited for teaching and development of a new Family Medicine curriculum for Botswana.

The 2011 Integrated Health Services Plan^11^ includes Family Medicine for the first time, and paves the way for Ministry of Health-funded posts for FMS and consultants (although specialist training is inaccurately still recorded as being OC or ‘Out of country’). Prior to 2007 Family Medicine was not part of the Botswana Human Resources for Health Plan,^17^ and was not even recognised as a specialty in the country. It was only after the 2008 workshop that the Botswana Health Professions Council added Family Medicine to the list of registered specialties. Family Medicine is relatively new to the continent of Africa. For example, Family Medicine was only recognised as a specialty by the Health Professions Council of South Africa in 2007.^18^

In January 2011 the first eight Batswana postgraduate doctors started the four-year MMed Fam Med training programme in Maun and Mahalapye. The second intake of postgraduates started training January 2012. Supervision and teaching of community placements for third- year undergraduates in the rural teaching complexes started in August 2011. This model of decentralised postgraduate training for Family Medicine will be evaluated more formally.

## Ethical considerations

This is a descriptive article developed by the authors in discussions about the future of Family Medicine in Botswana. There are no conflicts of interest or ethical considerations.

### Potential for Family Medicine in Botswana

The specialty of Family Medicine is practiced in different ways in different parts of the world, the role differing depending on clinical and resource contexts. Family Medicine is an entirely new specialty in Botswana. It is not surprising then that there is some lack of clarity about what can be expected from these new specialists, what their role is, and where they will be based. This article outlines some of the need and potential for these specialists in Family Medicine in Botswana from a public health as well as primary health care and Family Medicine perspective.

### Person-centred and patient-based care

First and foremost, the FMS will be providing a holistic approach to individuals and their families. They will be rooted in the community, preferably from the same area, and familiar with the customs, economy and epidemiology of the area.

FMS of the future in Botswana will be the first choice for medical assessment of any condition. They will be trained to a high standard across a wide range of specialties, as well as being trained in generic skills such as consultation and confident in clinical examination to elicit further information and reach a diagnosis. They will be well placed not only to deal with referrals from other members of the primary healthcare team but also to refer on to specialists and the appropriate level of care.5 

Worldwide, medical undergraduate training now emphasises problem-based learning. Postgraduates starting training to be specialists in Family Medicine in Botswana are no exception, wherever they did their undergraduate training. This discipline will stand them in good stead to source evidence-based medicine throughout their careers, even in remote and rural areas. The challenge is also to learn from patient-based care.

At present many Batswana patients are treated by separate clinics for HIV and AIDS, tuberculosis, antenatal care (ANC), maternal and child health (MCH) and non-communicable diseases (NCDs). This is time-consuming for patients and potentially dangerous if drug interactions occur unmonitored. Compliance declines with each additional ‘vertical programme’. An FMS is the best person to co-ordinate primary care programmes and integrate the care given. A clinical audit of patients seen in the Infectious Disease Control Clinic in Maun showed that 75% of the clients were also regularly attending at least one other clinic for NCDs, ANC, MCH or other problems.^19^ We will be studying this further, as we aim to transform these vertical programmes and health clinics into Family Medicine teaching and training sites with the help of a Medical Education Partnership Initiative grant.

### Community-orientated primary care 

The FMS with their knowledge and access to the community can identify common health problems and develop an appropriate intervention involving the community. They will be able to monitor the impact of the intervention as they will be providing continuity of care for the community as well as individuals – a skill no other hospital-based specialist will have. Those with MMed degrees in Family Medicine will be trained in and committed to developing community-oriented primary care.^15^ However, community-oriented primary care has not yet been implemented in Botswana, despite its appropriateness for a setting with a high proportion of the population (86%) living in remote and rural areas. This will be one of the opportunities and challenges facing Family Medicine.

The difficulties of implementing community-oriented primary care have been identified elsewhere.^6^ There is an inherent tension between person-centred care and the health needs of the community. In the northern hemisphere model of primary health care and Family Medicine, this tension is resolved by larger group practices with multi-disciplinary teams and partnerships with public health organisations and staff. 

### Access

Access is a major issue in most rural areas in Botswana, which results from a combination of distance and poverty. One immediate outcome of FMS working in primary hospitals and health clinics will be that patients and carers will be able to access care closer to home. This has been very popular elsewhere in the world, and improves patient satisfaction and access. FMS tend to see conditions early, when there is a better outcome from treatment. Care closer to home also reduces patient and carer travel time and costs, which also improves compliance with medication and follow up.

In the northern hemisphere model of Family Medicine there is a tension between access and FMS being the gateway to higher levels of health care, mainly for rationing (i.e. cutting costs). In Africa FMS are rarely the first point of contact, except perhaps in the private sector. Being a ‘gateway’ is less of an issue in pluralistic healthcare systems.

 The potential for Family Medicine Specialists in Botswana is to be able to treat 90% of the population closer to home, and refer on to other specialists in an appropriate and timely way. This aspect of access should be monitored regularly by the Family Medicine Specialists in order to audit their standards of care. 

### Acute care and emergencies

FMS will be trained to manage acute care and emergencies in district and primary hospitals. The Consensus Statement about Family Medicine in sub-Saharan Africa (SSA) identifies clearly that there is a need for Family Medicine doctors to be able to work in district and primary hospitals, whilst in remote areas they will need the skills to do emergency surgery.^4^

FMS in Botswana will be trained to have skills in surgery, obstetrics and gynaecology (such as assessment of fetal distress and lower-segment Caesarian section if necessary) (1) anaesthetics, (2) orthopaedics, (3) adult medicine, (4) paediatrics, (5) emergency medicine, (6) infectious disease control and (7) skins, eyes, ears, nose and throat and psychiatry. The Stellenbosch curriculum covers these 10 clinical domains, and the Family Medicine trainees will spend the first two years in the District General Hospitals in Maun and Mahalapye rotating through these specialties. They will then spend one year in the community, learning about primary care, and will complete the four-year training programme with a research dissertation and an elective period.

The aim is that more people will be treated appropriately and effectively in the primary or district hospital, and so take pressure off the major centres, especially Francistown and Gaborone. Reducing referrals to these major centres will be a measurable and high-priority outcome for Family Medicine. We will be setting up monitoring systems to see what happens to referral patterns from the FMS. It is of course possible that well trained, skilled doctors will identify more unmet needs and actually increase referral rates. We will be monitoring through audit and colleague collaboration the timeliness and appropriateness of referrals, and whether they were well managed up to the point of leaving the district hospital.

 At the same time, the FMS can use their training and clinical judgement by placing these emergencies within the context of their patients’ background. For example, when dealing with acutely ill children admitted with measles, they can identify whether this is because of failure of the immunisation programme at an individual and community level. They can also identify whether there are other children in the family at risk of contracting measles, and immunise preventatively.

The FMS will have access to a range of common and cost-effective laboratory tests, such as blood tests, thick films for malaria, immediate analysis of cerebrospinal fluid for bacteria and also cryptococci, HIV polymerase chain reaction, plain X-rays, etc. One task of the training programme will be to define a list of appropriate tests and interventions that all FMS should be familiar with and have reliable access to. Another task will be to define a list of essential drugs and equipment that should be available at each level of primary and community care.

### NCDs

FMS can improve the management of chronic diseases such as hypertension and diabetes. Stroke is common in Botswana, and a common cause of major morbidity (e.g. hemiparesis) in young people. A clinical audit done in Mahalapye (Rijken T 2010, personal communication) showed that the ‘Rule of Halves’ is still true in Botswana, where many other countries have improved the coverage and effectiveness of NCDs. (Notably in England and Wales since the New General Medical Services contract was introduced in 2004 with financial incentives to reach Quality and Outcomes Framework targets.) The Rule of Halves was first described by Tudor Hart,^21^ working in an isolated and deprived rural community in South Wales. He found by original community- based research and audit, that only half of the population with hypertension had ever had their blood pressure measured and recorded. Of the half that had hypertension recorded accurately, only half were on treatment. Of the half of the hypertensives who were on treatment, only half of them had adequately controlled blood pressure. This is a challenge for FMS working in the community and clinics, providing follow up and continuity of care closer to home. The numbers of people with hypertension needed to treat to prevent one stroke is eight. Management of hypertension is one of the most cost-effective interventions in all of our medical armamentarium, and yet we are still not doing it well, and thousands of young and old people in Botswana have severe, dense strokes that could have been prevented.

Family Medicine has a research grant as part of the Medical Education Partnership Initiative to transform the current vertical programmes in health clinics to integrated Family Medicine and primary care teaching sites. We will be reporting on this transformation to manage NCDs better in due course.

### Clinical governance and quality improvement

FMS will take a leading role in developing and setting quality standards and guidelines in Botswana. This has already started, with one of the trainers in Mahalapye developing Guidelines for the Management of Hypertension, Diabetes and Lower Abdominal Pain.

Funded by an international research grant from the Medical Education Partnership Initiative, a review is under way to adapt and collate a wide range of guidelines developed in Botswana to adapt, disseminate and implement primary care guidelines. Family Medicine is leading on this initiative, and will use the guidelines and the MMeds working in primary care and public health to transform health clinics in remote and rural areas into Family Medicine teaching and training sites. 

FMS at the interface between primary care and hospital medicine are ideally placed to take a leading role in training staff locally through continuing professional development, teaching and mentoring. Continuing professional development can become the local platform where all clinical staff are kept up to date in evidence-based medicine, where new guidelines are introduced locally and local audits and interventions are discussed to improve patient care.

Through these and other strategies, Family Medicine can help to develop district and primary hospitals and services to the highest possible standards of clinical care and local excellence. 

### Equity

By integrating non-communicable disease programmes into primary care, Family Medicine Specialists can improve equity of access to effective health care as well as improving quality. Botswana is a big country, with many people living far from the two major hospitals of Gaborone and Francistown. FMS will bring good health care to where the people are living, especially with the training programme already in Mahalapye and Maun, where training and teaching will be in the context of greatest healthcare need.

## Conclusion

These FMS of the future will graduate in Botswana in 2015. They will have had four years of carefully supervised training with ongoing assessment and examination. They in turn will be in a strong position to take on the roles of trainer, supervisor and mentor for others. The challenge for government, the Ministry of Health and University of Botswana is to make the training and subsequent posts created for Family Medicine appropriate and attractive, so that this new cadre of specialists will stay in Botswana and take their place delivering high-quality health care where it is most needed. They will be well placed to take over the teaching and training programmes for both undergraduates and postgraduates in Family Medicine, and be inspirational leaders for future students in Botswana.

Graduates of the University of Botswana MMed Family Medicine degree course will be highly trained and skilled doctors who can work in primary or district hospitals, rural and urban clinics, and also go into private practice with high standards and a working knowledge of Ministry of Health facilities and resources.

This is our vision of Family Medicine in Botswana, that we are sharing with the first eight postgraduates who started the MMed Family Medicine in January 2011.

We hope that this provides an outline of the potential for Family Medicine in Botswana, and can be of use in thinking about the numbers and distribution of FMS in the country’s Integrated Service Plan for Health.

## References

[1] World Health Organisation. Primary Health Care: Now More than Ever. In WHO. World Health Report 2008. Geneva: WHO; 2008. Available from: http://www.who.iny/whr

[2] World Bank. World Development Report: Investing in Health. New York: Oxford University Press; 1993

[3] Ssenyongs R, Seremba E Family Medicine’s Role in Health Care Systems in Sub-Saharan Africa: Uganda as an Example. Fam Med 2007;39 (9):623–626 17932794

[4] Mash R, Reid S Statement of Consensus on Family Medicine in Africa. Afr J Prim Health Care Fam Med 2010;2 (1)

[5] Mash R, Downing R, Moosa S, de Maeseneer J Exploring the key principles of Family Medicine in sub-Saharan Africa: international Delphi consensus process. SA Fam Pract 2008;50 (3):60–65

[6] Reid SJ, Mash R, Downing RV, Moosa S Perspectives on Key Principles of Generalist Medical Practice in Public Service in Sub-Saharan Africa: a Qualitative Study. BMC Family Practice 2011;12:67 21726454 10.1186/1471-2296-12-67 PMC3142501

[7] Colleges of Medicine South Africa. [Homepage on the internet]. [cited 2010]. Available from: http://www.collegemedsa.ac.za

[8] Jenkins L, Mash B, Derese A Development of a portfolio of learning for postgraduate family medicine training in South Africa: a Delphi study. BMC Family Practice 2012;13:11 22385468 10.1186/1471-2296-13-11 PMC3317832

[9] Government of Botswana. Central Statistics Office. [Homepage on the internet]. [cited 2011]. Available from: http://www.gov.bw

[10] Good K Diamonds, Dispossession and Democracy in Botswana. Johannesburg: Jacana Press; 2008

[11] University of Botswana, Faculty of Health Sciences, School of Medicine Proposal for the Introduction of Master of Medicine in Anaesthesia, Emergency Medicine, Family Medicine and Public Health in addition to Master of Internal Medicine and Paediatrics established in January 2010. Gaborone: University of Botswana; 2011

[12] Ministry of Health, Government of Botswana Integrated Health Service Plan: A Strategy for Changing the Health Sector for a Healthy Botswana 2010–2020. Ministry of Health; 2010

[13] Botswana Long Term Vision Council. Vision 2016: Long term Vision for Botswana. [Homepage on the internet]. [cited 2011]. Available from: http://www.vision2016.co.bw

[14] Rhyne R, Bogue R, Kukulka G, Fulmer H, editors. C. COCP. Washington DC: American Public Health Association; 1998

[15] Pfaff C Report of the Feasibility of Family Medicine Training in Letsholathebe 2 Memorial Hospital in Maun. Gaborone: University of Botswana; June 2010

[16] Rijken T Report of the Feasibility of Family Medicine Training in Mahalapye District Hospital. Gaborone: University of Botswana; September 2010

[17] Ministry of Health. Human Resources Plan 2010-2020. Gaborone: Ministry of Health

[18] Thupayagale–Tshweneagae G Migration of Nurses in Botswana. A study for the BTPP/ BIDPA in conjunction with the Nurses Association of Botswana. Gaborone: Department of Nursing Education, University of Botswana; 2006

[19] Hellenberg D, Gibbs T Developing Family Medicine in South Africa: A new and important step for medical education. Medical Teacher 2007;29:897–900 18158661 10.1080/01421590701827890

[20] Parsons L Audit of Co-morbidity in IDCC in Maun. Gaborone: University of Botswana; 2010

[21] Hart JT ‘Rule of Halves’: Implications of increasing diagnosis and reducing drop out for future workload and prescribing costs in primary care. Br J Gen Pract 1992;24 (356) PMC1371996 1493028

